# Marijuana policy and tribal communities in the United States

**DOI:** 10.1057/s41271-025-00572-y

**Published:** 2025-06-01

**Authors:** Daphne E. Pedersen

**Affiliations:** University of North Dakota, Grand Forks, ND USA

**Keywords:** American Indian and Alaska Native, Cannabis, Marijuana policy, Native Americans, Reservations, Tribal health

## Abstract

In the United States (US), the policy landscape surrounding marijuana is complex, multijurisdictional, and often messy, if not contradictory—particularly for tribal communities. Currently, tribes may choose to criminalize or legalize marijuana but may be located within a state or adjacent to a city that has opposing policies. With patterns of substance use that are notably different from the US population as a whole, including higher rates of marijuana use and dependence among Native American youth, tribal communities have important policy decisions to make that will directly impact public health. This paper reviews the history and policy background related to marijuana in the United States, associated health concerns for American Indian and Alaska Native communities, and considerations for tribal communities seeking how to best move forward. A community-led public health response that is culturally grounded can more effectively promote Indigenous health and sovereignty worldwide.

## Key message

- Federal, state, and tribal marijuana policies are frequently at odds, leaving tribal governments with a complex web of regulations to navigate as they determine the most appropriate course of action for community members.
- Tribal health and well-being are particularly sensitive to changes in marijuana legalization, with minors most at risk despite age-related regulations for recreational marijuana use.
- Tribes are encouraged to carefully weigh decisions regarding legalization and cannabis markets, and to create a tribal health code that prioritizes public health.

## Introduction and policy background

In the United States, the policy landscape surrounding marijuana is complex, multijurisdictional, and often messy, if not contradictory—particularly for tribal communities. Considered legal under federal law until the Marihuana Tax Act of 1937, possession of marijuana was banned by many states around the same time [1]. In 1951, the Boggs Act created mandatory prison sentences for specific drug offenses including marijuana. This was followed by the Narcotic Control Act of 1956, which increased penalties for drug offenses. In the 1960s, the constitutional basis for drug control shifted away from taxation to regulation of interstate commerce. At this time, responsibility moved from the Treasury to the Department of Justice, coinciding with President Nixon’s “war on drugs”[1].

Working with Congress, Nixon increased federal drug control through a series of legislative acts including the Controlled Substances Act (CSA) of 1970, which continues to serve as the foundational federal policy regarding marijuana. The CSA orders drugs based on their addictive properties and dangerousness, including risk to public health. Whereas Schedule V drugs are the least addictive and dangerous, Schedule I drugs are believed to have “high potential for abuse” and “no currently accepted medical use in treatment in the United States.”[1] As such, they are considered illegal to produce or manufacture, distribute, dispense, or possess—either for medical or personal/recreational purposes [1]. Under the CSA, marijuana and its derivatives are classified as Schedule I drugs making marijuana the most used illicit drug in the United States [2].

Several actions have been taken in recent years to both affirm the CSA and hinder its enforcement. Following a directive from former President Obama to the Department of Justice, in states where marijuana has been legalized for medicinal or recreational use, no federal funds are directed to the prosecution of marijuana-related crimes if terms of the Cole Memorandum are met. This document outlines eight enforcement priorities, including prevention of distribution to minors [3]. Later, a memorandum of understanding was sent clarifying the policy of federal non-interference, indicating that the same considerations would apply to all federally recognized reservations. As noted in the memorandum, “the eight priorities in the Cole Memorandum will guide United States Attorneys’ marijuana enforcement efforts in Indian Country, including in the event that sovereign Indian Nations seek to legalize the cultivation or use of marijuana in Indian Country”[4]. Thus, neither the Cole Memorandum nor the memorandum of understanding to tribal leaders altered the legal status of marijuana on reservations, nor did it remove the authority of federal law enforcement on reservations in cases where public safety is compromised [5].

During the first Trump administration, the Marijuana Enforcement Memorandum rescinded the policy of non-interference and granted federal prosecutors the authority to determine how to prioritize enforcement of federal policy [6]. US Attorneys are expected to “weigh all relevant considerations, including federal law enforcement priorities set by the Attorney General, the seriousness of the crime, the deterrent effect of criminal prosecution, and the cumulative impact of particular crimes on the community”[7]. The directive again allows for federal resources to be used for marijuana enforcement [8]. Although the Department of Justice declared that it will not focus on individual offenders or state-authorized marijuana industries, the Memorandum reaffirmed that the growth and trafficking of marijuana remain federal crimes that can be prosecuted. Thus, the extension of the Cole provisions to tribal governments was effectively repealed [1].

Reflecting the priorities and position of each administration, federal policy has remained in flux. In 2021 President Biden announced that he would pardon prior federal offenses that involved simple possession of marijuana and urged governors to do the same for state-level offenses. He also asked that administrative review of marijuana as a Schedule I drug be undertaken by the Departments of Health and Human Services and Justice. If marijuana was reclassified within the CSA, resources could be channeled to the Food and Drug Administration for the management and assessment of marijuana and cannabis products [1]. While Biden’s directives allow greater leniency for adult and medicinal use of marijuana and cannabis products, he maintained that limitations on trafficking, marketing, and sales to minors remain [9]. To date, the Cole Memorandum has not been revisited.

As Schedule I drugs, marijuana and cannabis products (with the exception of hemp plant material) are meant to be strictly regulated at the federal level. Yet, policy actions taken over the last several years at the federal, state, city, and tribal levels present an inconsistent policy landscape. Today, 38 US states, three territories, and the District of Columbia have legalized cannabis products for medical use. Twenty-four states, two territories, and the District of Columbia have measures that allow for adult recreational use of marijuana. In 11 states, residents can be jailed for possession of marijuana [6]. State-level initiatives generally either legalize or decriminalize possession of a specific quantity of marijuana or cannabis products among adults aged 21 and over, and some provide a state-administered regulatory strategy for sales. To date, no state has reversed the decision to legalize either medical or recreational use of the drug [1].

To complicate matters, just as there are discrepancies between state and federal policies, efforts of some cities to decriminalize marijuana run counter to state and federal mandates [1]. This is also the case for some tribes that may choose to criminalize or legalize marijuana but are located within a state or adjacent to a city that has opposing policies. On the Pine Ridge reservation in South Dakota, for example, marijuana is legal but alcohol sales, possession, and consumption are not [10]. Recreational marijuana remains illegal in the state of South Dakota, although medical marijuana use is allowed; someone who purchases cannabis products on the reservation risks arrest once they leave if they do not have a South Dakota medical marijuana card [11]. The owner of the single dispensary in Pine Ridge has estimated that half of its customers are not from the reservation, coming instead from other parts of the state and neighboring Nebraska [12].

For reservation populations grappling with marijuana policies, it is still unclear whether federal or state laws prevail. Although tribal nations are considered to be sovereign, in many states Public Law 280, a federal law enacted by Congress, transfers federal authority in civil and criminal cases on tribal lands to the state. This allows even minor crimes to be prosecuted if part of the state’s criminal law. South Dakota is not one of the five states under Public Law 280 that has been granted jurisdiction over tribal members [13], but in those states in which Public Law 280 applies, there is immense uncertainty when it comes to both tribal law and marijuana law [14].

Based on the principles of federalism, state and tribal laws regarding the sale, distribution, and possession of marijuana and low-THC (tetrahydrocannabinol) cannabis products are preempted by federal policy and the federal government can choose to enforce the CSA if it so chooses [1]. And although federally recognized tribes are considered sovereign nations, Congress retains the authority to legislate on tribal issues. The federal government has the “right to challenge a state marijuana law if that state does not implement strong and effective regulatory and enforcement systems that will address the threat that state law could pose to public safety, public health, and other law enforcement interests…. These considerations and limitations apply in Indian Country.”[5] Thus, even with state support, tribes may still be subject to federal interference.

Protections for state and territorial marijuana markets are regulated by Congress, but tribes have not been included in those policies, and many states lack a formal policy to allow tribes to participate in statewide cannabis sales, regulation, and taxation strategies [12]. Some states have developed tribal-state compacts to govern marijuana production and sales on tribal lands. Under compact agreements, sharing of tax revenue is used to secure protection from prosecution through inclusion in state cannabis programs, which are protected by Congress [15]. The Suquamish and Squaxin Island Tribes, for example, are two of the 22 federally recognized tribes within Washington state that have signed compacts allowing them to grow, process, and/or engage in distribution of cannabis. Consistent with state policy, marijuana remains age-restricted and must be purchased from a state-licensed or tribal store.

Many of the remaining tribes within Washington are also in the process of negotiating compacts, and Washington currently has the largest number of tribally owned and operated dispensaries. The Yakama Nation is an exception; the tribe ruled against recreational marijuana in 2014 and continues to ban liquor and cannabis sales on tribal lands including all 10 counties making up the reservation [16]. In this case, the criminalization of marijuana within a state where it is legal creates concerns about enforcement and public health. In other cases, this concern works in reverse—a tribe has legalized recreational cannabis use but exists within a state with more restrictive policies. The Eastern Band of Cherokee Indians in western North Carolina first decriminalized possession of marijuana in 2021. In 2023, recreational use of marijuana was approved by the tribal council and a dispensary was opened on tribal lands. Recreational use of marijuana remains illegal within the state of North Carolina and attempts to legalize medical cannabis have failed. Although North Carolina is sometimes cited as a state in which decriminalization has occurred, possession of small amounts of marijuana remains a misdemeanor offense [17]. Despite lack of clarity and a contradictory policy environment, the number of tribally owned dispensaries is rapidly increasing. Currently, tribes from at least nine states (California, Michigan, Minnesota, Nevada, New Mexico, New York, North Carolina, South Dakota, and Washington) operate cannabis dispensaries.

### American Indian and Alaska Native health outcomes

Patterns of substance use are notably different in American Indian and Alaska Native communities compared to the US population. While lifetime rates of alcohol and drug use, including marijuana, are lower in some communities given high rates of abstinence, substance use disorders are more common and early use of substances is striking [18]. Rates of marijuana use and dependence are higher among Native Americans, including youth, than US rates, and are higher than any other racial or ethnic group [19–21]. American Indian youth who reside on reservations are at disproportionate risk [22]. In a study of two reservation communities, Native youth were more likely than their same-age peers to use marijuana and did so at an earlier age [23]. Among American Indian eighth graders, nearly 40% report having used marijuana in the last 30 days [24]. Examination of national data indicates that substance use among American Indian eighth graders is “dramatically higher” than national averages and lifetime use rates approach 50 percent [20].

Problematically, there is a link between adverse childhood experiences and both early and later drug use: those who suffer higher rates of trauma, abuse, violence, and loss are more likely to engage in alcohol and drug use. In turn, because of early experiences, prevention and intervention efforts are less effective [25, 26]. Compared to the general population, American Indian youth have a higher risk profile, having lived through more of these troubling experiences [27]. Marijuana has been described as a significant gateway drug for reservation populations, especially when alcohol is illegal [23], and it is linked with future substance abuse disorders among Native youth [28, 29]. Early and heavy marijuana use is associated with lower motivation and task performance, diminished affective and cognitive response, neurocognitive issues and slowed brain development, and a variety of outcomes linked to future potential such as dropping out of school, unemployment, and poverty [2, 30, 31]. The maldistribution of problems related to substance use on reservations, including poor academic performance and high drop-out rates, delinquency, crime and violence, suicidality, and alcohol-related deaths, suggest that there is a heavy price to be paid by tribal populations [20].

Noted by the US Department of Justice [5], many tribes have expressed concern about the potential public health and safety impacts of marijuana legalization by the states given existing problems with substance use, illegal cultivation of marijuana on reservation lands, gang activity, and crimes related to the drug trade. Studies indicate that since legalization, perceptions of harm have been declining and recreational use of marijuana has been increasing, including among minors [32, 33]. Coupled with the unique reservation living environment and high rates of use, prevention of and treatment for substance abuse is challenging [24]. Thus, a related public health concern is the potential impact that legalization of marijuana has on adolescent use in the context of social learning and observation. Prevention efforts are challenged when youth live in households where adults and siblings may be using marijuana, some based on claims of its medical and treatment potential [34]. Given its status as a Schedule I drug, empirical evidence of marijuana’s legitimate medicinal benefits is limited to only a few conditions [35]. Despite limited scientific testing, the association between perceptions of risk and both intentions to use and frequency of marijuana use among American Indian adolescents [36] indicates a probable link between family member and household use, a reduction in perceptions of marijuana’s risk, and adolescent use.

In advising tribes considering marijuana legalization, public health should be the primary concern [37], particularly protection of the most vulnerable populations. Emerging evidence suggests that since legalization, marijuana use among adolescents and young adults has increased; it is difficult to fully understand the downstream effects of marijuana legalization on pediatric and adolescent health at this time [38]. Among adults, negative outcomes include an increase in motor vehicle accidents, trauma-related cases, hospital and emergency service utilization, and adverse birth outcomes including low birth weight [39]. There is also concern that the public health impacts of increased marijuana use will be seen in exacerbation of existing mental and physical health issues (schizophrenia, psychosis, chronic bronchitis, asthma, pulmonary disorders, etc.), unwanted or accidental exposure among young children, and increased use of other substances like opioids [35].

### Recommendations

Although great uncertainty exists in the policy and enforcement landscapes, it does appear that the legalization of marijuana for medicinal and/or recreational use will not be reversed in US states and there is movement among many tribes to legalize as well. Cannabis production is a lucrative industry that may afford tribes a new source of economic growth, raising the standard of living among reservation populations [15] and allowing tribes to address underfunding issues in infrastructure, housing, and public education [40]. Yet, marijuana does represent a threat to public health, particularly among youth. Although differences exist across cultural and value-based systems, and some tribes view marijuana as a natural product that comes from the environment [10], the empirical evidence is clear that early and routine marijuana use, especially among youth, is tied to negative health outcomes and reduced social and economic life chances including poverty and disadvantage in adulthood [2, 30, 31]. Substance use among American Indian and Alaska Native youth is consistently high [41]. Following legalization, daily use rates and marijuana vaping have increased significantly among US adolescents generally [19]. Thus, we can expect that as marijuana becomes legal on more reservations, already high rates of use may be magnified among Native American youth.

Clearly, there are advantages and disadvantages that tribes must consider related to legalization and the decision whether to engage in production and sales. Benefits include, but are not limited to: (a) economic development, allowing for investment in tribal infrastructure and programming; (b) establishment of safe production systems and greater regulation of products through enforcement of manufacturing standards; (c) transparency of product information, including THC (tetrahydrocannabinol) content, for consumers; (d) reduced strain on correctional systems due to decriminalization of drug-related offenses; and (e) potential reduction in opioid use (when used as an alternative for chronic pain management) and overdose deaths due to drug lacing. At the same time, significant drawbacks to legalization and production/distribution include: (a) increased access and use among minors despite policies restricting possession and sales; (b) increased traffic on tribal lands where dispensaries are located; (c) downstream health effects among routine users; (d) worsening of mental health symptoms among some users with a history of depression and trauma; and (e) potential increase in opioid and other drug use [42].

Thoughtful and careful action is needed moving forward. For tribes considering whether to enter production, distribution, and sales markets, several factors are relevant. These include the need to create policies at the tribal level, potential for state and federal prosecution, investment funding, banking options, the need for security and law enforcement, restrictions of the Indian Gaming Regulatory Act related to use of gaming profits, taxation by the state, potential risks and benefits for the community, and consequences for the reservation environment [43]. Implicit in the impact to the tribal community is consideration of its health and wellbeing. Other tribes that choose not to legalize marijuana will need to determine how to live within a contradictory policy environment that may inadvertently result in greater access to marijuana and potential trafficking on reservation lands despite tribal policies to the contrary. For all reservations, where marijuana is legal and where it is not, it is likely that increased law enforcement will be needed to help target and prevent access among minors. Furthermore, clear communication between tribal leaders and the state and federal governments is needed to ensure that the position and interests of the tribe are well understood and protected.

It is also important that tribes understand and articulate their own position and interests as a collective. Involvement of all tribal members with respected interests in the life, values, and priorities of the community and a close awareness of its strengths and potential challenges, should be a priority. The values and concerns of the tribal community related to marijuana and cannabis should be writ into both the tribal code and a tribal health code. Because many still lack a health code, development and/or restructuring of these policies will take significant effort and time.

## Conclusions

Tribal communities in the US will need to remain vigilant and nimble, as marijuana policy is rapidly evolving. Constant attention to the policy and public health landscapes is needed to ensure the health and safety of community members. As marijuana access increases, whether by legalization on or off tribal lands, tribes will need to monitor emerging issues and be prepared to develop and implement responsive and culturally appropriate strategies to address potential challenges. Working with partners within the tribal community as well as at the local, regional, state, and federal levels is critical. Developing an awareness of the history and policies related to marijuana in the US provides necessary scaffolding for public health leaders navigating complex social, economic, and legal contexts in American Indian and Alaska Native communities responding to change. This background offers critical insight into how colonization, regulatory frameworks, and decades of systemic inequality have converged to shape the wellbeing of American Indian and Alaska Native peoples today. An informed public health response that is sensitive to community history and current social and economic challenges can better address issues of access to care, substance use and abuse, and the trajectories and future outcomes for youth. For the global health community, this understanding reinforces the need for culturally grounded, community-driven approaches and emphasizes the wider impact of drug policy on Indigenous health and sovereignty around the world.
